# Effect of Temperature and Covering Structures in Seed Dormancy and Germination Traits of Manchurian Striped Maple (*Acer tegmentosum* Maxim.) Native to Northeast Asia

**DOI:** 10.3390/plants14050767

**Published:** 2025-03-02

**Authors:** Sieun Kim, Chung Ho Ko, Hak Cheol Kwon, Yong Ha Rhie, Seung Youn Lee

**Affiliations:** 1 Division of Wild Plant and Seed, Baekdudaegan National Arboretum, Bonghwa 36209, Republic of Korea; sekim@koagi.or.kr; 2 Department of Horticulture and Breeding, Graduate School of Andong National University, Andong 36792, Republic of Korea; 3 Garden and Plant Resources Division, Korea National Arboretum, Yangpyeong 12519, Republic of Korea; tune0820@korea.kr; 4 Natural Product Informatics Research Center, Korea Institute of Science and Technology, Gangneung 25451, Republic of Korea; hkwon@kist.re.kr; 5 Department of Interdisciplinary Program in Smart Agriculture, Kangwon National University, Chuncheon 24341, Republic of Korea; 6 Department of Horticulture, Kangwon National University, Chuncheon 24341, Republic of Korea; 7 Department of Smart Horticultural Science, Andong National University, Andong 36729, Republic of Korea

**Keywords:** cold stratification, deep physiological dormancy, mechanical resistance, move-along experiment, samara

## Abstract

*Acer tegmentosum*, an indigenous medicinal plant under threat from overexploitation, is a deciduous tree species native to Northeast China, southern regions of the Russian Far East, and Korea. In this study, we analyzed the characteristics of samaras (single-seeded fruit) of *A. tegmentosum* to determine the type of seed dormancy as well as to identify the factors responsible for dormancy release. We identified the seed dormancy to be that of deep physiological dormancy (PD). PD can be combined with mechanical resistance of the seed coat, which limits the protrusion of the radicle during germination. We observed that mechanical resistance exhibited by the water-permeable testa is associated with PD of *A. tegmentosum*. This was previously attributed to seed dormancy in *Acer* as testa-imposed dormancy or embryo dormancy. In *A. tegmentosum*, PD and mechanical resistance of the testa were overcome through cold stratification treatment at 1 and 4 °C, which was similar to winter duration under natural conditions. The pericarp of samaras facilitated germination at an early spring temperature (15/6 °C) after cold stratification at 1 and 4 °C, enabling the seedling survival of *A. tegmentosum*. We concluded that the covering structures composed of testa and pericarp in *A. tegmentosum* play vital roles in dormancy release and subsequent seed germination; they respond to external environmental cues based on the climatic conditions of Northeast Asia. This adaptation probably determines their behavior at an early life stage in response to environmental factors.

## 1. Introduction

*Acer tegmentosum* Maxim., commonly known as Manchurian striped maple, belongs to the section *Macrantha* and is a deciduous tree native to Northeast China, southern regions of the Russian Far East, and Korea [1]. *A. tegmentosum* in Korea is distributed from 605 to 1413 m above sea level in Gangwon province and at Mt. Jirisan; individual trees grown from seeds are observed in the canopy and understory of broadleaf deciduous forests [2,3]. This woody plant has an upright stem and bright white stripes on its green-gray bark, giving it excellent ornamental value. It is also known to have strong disease and insect resistance, making it a very promising plant as an ornamental plant [4]. In Korea, it is locally known as “Beolnamu” or “Sancheong-mok” and is traditionally used to treat liver diseases and leukemia [5,6]. However, the indiscriminate collection of *A. tegmentosum* for medicinal purposes has restricted its habitat to inaccessible valleys and made the population distribution independent rather than clustered [3]. *A. tegmentosum* is likely to be extinct due to its indiscriminate and illegal collection [7]; it is considered as a species of least concern (LC) by the IUCN [8], whereas the Chinese species red list considers it to be vulnerable (VU) [9]. Moreover, population decline of the vulnerable species, *Rosalia coelestis* Semenov, is related to the indiscriminate collection of their preferred host plant, *A. tegmentosum* [10,11].

*A. tegmentosum* belongs to the northern lineage plants [12], which migrated from the north to the Korean Peninsula during the glacial periods of the Pleistocene epoch and were isolated in the high altitudes or algific talus slopes during the interglacials and Holocene [2,12,13]. It was reported that northern lineage plants, which are mainly distributed in mountainous areas, are predicted to be threatened by climate change if they cannot migrate to new habitats [14]. Seeds are used in the conservation of threatened native plants; this includes both in situ conservation through the direct seeding of collected seeds and the reintroduction of seedlings produced through ex situ conservation [15]. Although seed dormancy is a delaying mechanism that aids long-term survival of seeds in the natural environment, the proper understanding of seed dormancy is essential to avoid problems during conservation [16,17]. In addition to conservation, information on seed dormancy and germination traits would help understand the ecology of plants in their habitats [17,18].

Baskin and Baskin [19] categorized seed dormancy into five classes: physiological dormancy (PD), morphological dormancy (MD), morphophysiological dormancy (MPD), physical dormancy (PY), and combinational dormancy (PY + PD); PD is further divided into three levels: deep, intermediate, and non-deep. Mature seeds of the genus *Acer* are known to predominantly exhibit PD at dispersal from the mother plant [20]. Induction and breaking of PD are not controlled by absolute amounts of phytohormones, but the balance of endogenous abscisic acid (ABA):gibberellin (GA) levels and sensitivity are important for dormancy controls [21]. GA biosynthesis is known to be affected by environmental stimuli such as light or chilling [22]. In natural environments, although seeds with PD require specific conditions to break dormancy, artificial treatments such as stratification or after-ripening can be a substitute for natural conditions [20]. For example, the specific temperature range (0–10 °C) is used for cold stratification, and seeds of *A. platanoides* showed that dormancy release is more advantageous at lower temperatures even within the optimum temperature range for cold stratification [20,23].

In some *Acer* species, seeds with PD require cold stratification rather than warm followed by cold stratification to break the dormancy [24,25]. Warm stratification at 20 °C followed by cold stratification at 5 °C reduced the level of ABA in *A. saccharum* seeds; dormancy was only broken through cold stratification, which led to a decrease in ABA levels and an increase in the accumulation of GA-like substances [26]. Cold stratification at 5 °C for 12 weeks significantly reduced the ABA concentration of *A. morrisonense* seeds; bioactive GAs was detected during cold stratification [27]. Chen et al. [27] proposed that the decrease in the ABA:GA ratio was related to breaking the seed dormancy in *A. morrisonense*. *A. platanoides* seeds with deep PD exhibited a decrease in the accumulation of the transcription factor ABI5 induced by ABA through cold stratification at 3 °C [28]. Thus, it seems possible that cold stratification can break the PD of *Acer* species by reducing the ABA (germination inhibitor):GA (germination promoter) ratio in embryos. On the other hand, cytokinins such as 6-benzylaminopurine and kinetin are known to affect seed dormancy in *Acer* [29,30].

Nevertheless, requirements for germination and life cycle progression vary with species; some species do not require seed dormancy to delay germination and may germinate right after dispersal [31]. Germination of *A. saccharum* subsp. *skutchii*, distributed in the cloud forests of Mexico and Guatemala, was not promoted by cold stratification at 4 to 5 °C for 30 days [32]. *A. saccharum* subsp. *skutchii* has non-dormant seeds at dispersal time [20]. In contrast, cold stratification similar to that observed during winter is necessary to break the dormancy and promote the germination of *A. saccharum*, which is distributed in the cool, moist regions of North America [33,34]. Each species may vary in germination traits due to phylogeny, geographic distribution, habitat, and life cycle [35]. Therefore, identifying commonalities and differences between each species within the same genus may help understand the germination ecophysiology of *Acer*.

Understanding seed dormancy and germination traits with respect to ecological factors is essential for the conservation and ornamental utilization of *A. tegmentosum* genetic resources. Although previous studies indicated that outdoor stratification from autumn to spring and cold stratification could break their seed dormancy [36,37], the class of seed dormancy and germination traits of *A. tegmentosum* are yet to be determined. Thus, the objectives of this study were to determine (1) the class of seed dormancy and establish the relationship among seed dormancy, germination, and environmental factors, (2) the optimum cold stratification period and temperature required to break seed dormancy, (3) the effects of exogenous GA_3_ on breaking seed dormancy, (4) the phenology of seed germination and seedling emergence under near-natural temperature conditions, and (5) the cause of pericarp- and testa-imposed dormancy in *A. tegmentosum*. A series of experiments would help clarify the germination ecophysiology of *A. tegmentosum*.

## 2. Results

### 2.1. Samara Characteristics

**Figure 1:**
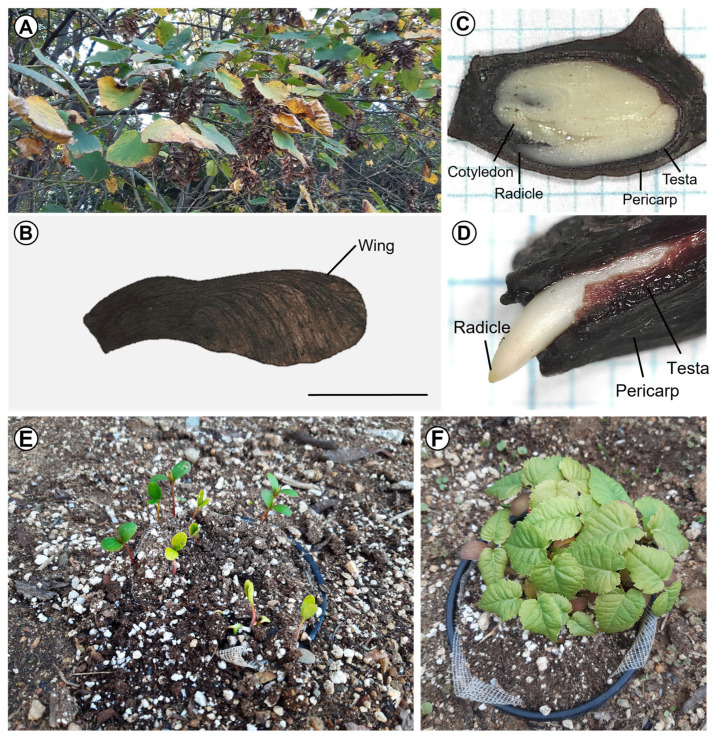
Samaras (single-seeded fruit) and seedlings of *Acer tegmentosum*. (**A**) Clusters of mature samaras on the branches of *A. tegmentosum*; (**B**) a mature samara of *A. tegmentosum*; (**C**) longitudinal section of an *A. tegmentosum* samara; (**D**) close-up of radicle emergence from an *A. tegmentosum* samara; (**E**) seedling emergence of *A. tegmentosum*; (**F**) *A. tegmentosum* seedlings at first leaf stage. Scale bar indicates 1 cm.

**Figure 2:**
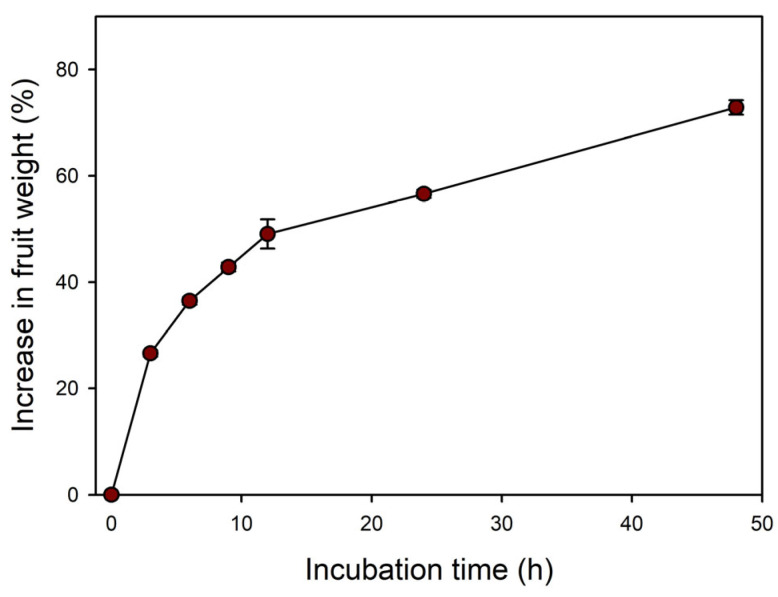
Imbibition of *Acer tegmentosum* samaras (single-seeded fruit) incubated for 48 h on moistened filter paper. Vertical bars represent SE (n = 4).

The length, width, thickness, and 100 seeds’ weight of samaras were 10.4 ± 0.12, 5.2 ± 0.08, 2.1 ± 0.04 (mm), and 3.9 ± 0.03 (g), respectively (Table 1). The internal morphology of *A. tegmentosum* samaras showed a fully developed bent embryo (Figure 1C). In the imbibition test, the increased weight after 48 h of samaras was 72.9 ± 1.34 (Figure 2).

### 2.2. Phenology of Germination and Seedling Emergence in the Field

**Figure 3:**
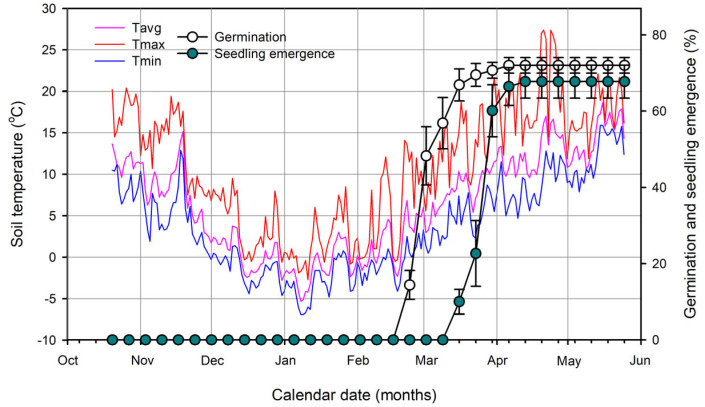
Cumulative germination and seedling emergence of *Acer tegmentosum* samaras (single-seeded fruit) in the field. Tavg, Tmax, and Tmin indicate daily average, maximum, and minimum soil temperatures, respectively. Vertical bars represent SE (n = 4).

The initiation of pericarp-testa rupture and radicle protrusion in *A. tegmentosum* samaras was observed in February (Figure 1D and Figure 3). Samaras germinated to 70.7 ± 2.08% at the end of March. The mean of the daily maximum temperatures and daily minimum temperatures during March was 13 ± 0.73 and 4.4 ± 0.4 °C, respectively. The final percentage of germination and seedling emergence was 72 ± 2.02 and 68.2 ± 4.19%, respectively (Figure 3). Seedling emergence of *A. tegmentosum* showed phanerocotylar epigeal germination (Figure 1E,F).

### 2.3. Move-Along Experiments

**Figure 4:**
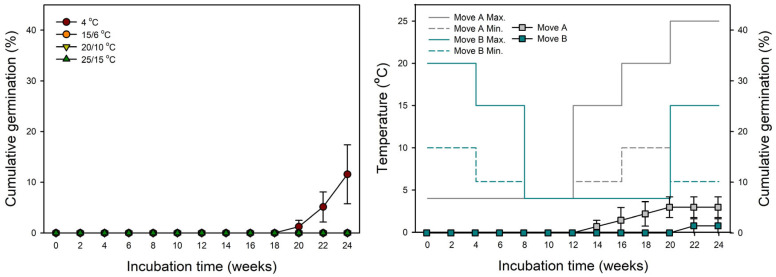
Cumulative germination of *Acer tegmentosum* samaras (single-seeded fruit) affected by move-along experiments. Colored solid and dashed lines indicate maximum and minimum temperatures, respectively. Vertical bars represent SE (n = 4).

Samaras germinated to 11.6 ± 5.82% after incubation at 4 °C for 24 weeks. In contrast, germination was not observed at 15/6 °C, 20/10 °C, or 25/15 °C for 24 weeks. Samaras in Move A germinated to 5.1 ± 2.04%, but samaras in Move B germinated to 1.4 ± 1.39% after incubation for 24 weeks (Figure 4).

### 2.4. Effect of Cold Stratification on Germination

**Figure 5:**
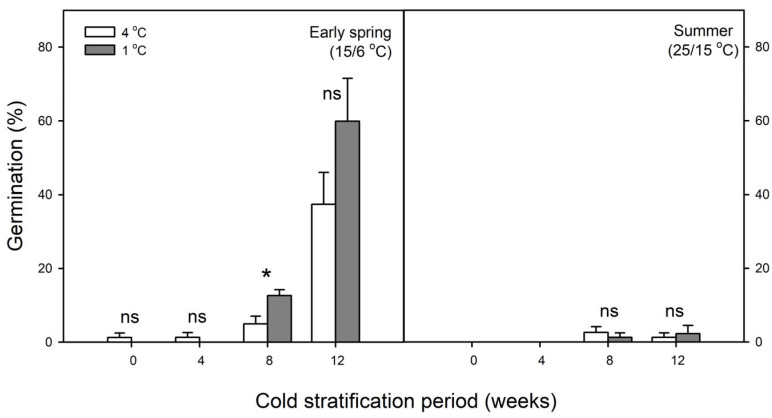
Effect of cold stratification at 1 °C and 4 °C on germination of *Acer tegmentosum* samaras (single-seeded fruit) after incubation at 15/6 °C and 25/15 °C for 14 weeks; ns and * indicate non-significant and significant results at *p* < 0.05 between cold stratification at 1 °C and 4 °C, respectively. Vertical bars represent SE (n = 4).

After cold stratification at 1 and 4 °C for 0 and 4 weeks, samaras germinated to ≤5% after incubation at 15/6 °C for 14 weeks. After cold stratification at 1 and 4 °C for 8 weeks, samaras germinated to 12.7 ± 1.57 and 5 ± 2.04% after incubation at 15/6 °C for 14 weeks, respectively. Germination after cold stratification at 1 °C for 8 weeks was significantly higher than that after cold stratification at 4 °C for 8 weeks (Figure 5). After cold stratification at 1 and 4 °C for 12 weeks, samaras reached 59.9 ± 11.63% and 37.4 ± 8.63% germination after incubation at 15/6 °C for 14 weeks, respectively. However, samaras germinated to ≤5% at 25/15 °C after cold stratification (Figure 5).

### 2.5. Effect of Exogenous GA_3_ on Germination

**Figure 6:**
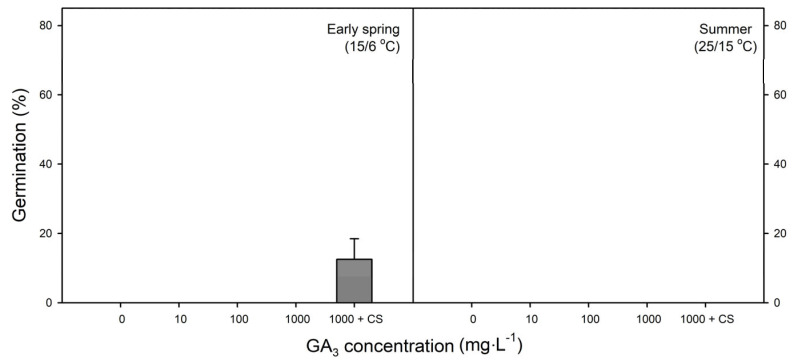
Effect of GA_3_ on germination of *Acer tegmentosum* samaras (single-seeded fruit) incubated at 15/6 °C and 25/15 °C for 14 weeks; 1000 + CS indicates treatment of 1000 mg∙L^−1^ GA_3_ followed by cold stratification at 4 °C for 12 weeks. Vertical bars represent SE (n = 4).

None of the samaras germinated after the 14-week incubation period in all concentrations of GA_3_ treatments. In the combination treatment of GA_3_ (1000 mg∙L^−1^) and cold stratification, samaras germinated to 12.5 ± 5.95% after incubation at 15/6 °C for 14 weeks, but no samaras germinated during incubation at 25/15 °C for 14 weeks (Figure 6). Therefore, the single treatment with GA_3_ was ineffective in breaking the dormancy of samaras.

### 2.6. Effect of Pericarp and Testa on Germination

**Figure 7:**
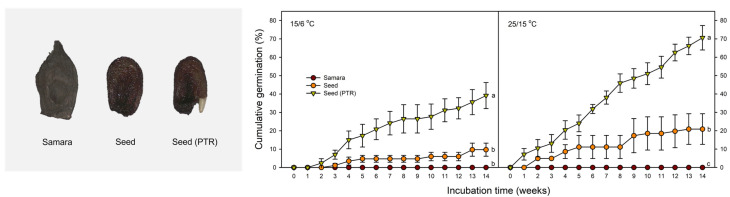
Effect of pericarp and testa on cumulative germination of *Acer tegmentosum* samaras (single-seeded fruit) and seeds incubated at 15/6 °C and 25/15 °C for 14 weeks. ‘Seed’ indicates that the pericarp was removed from the samara (single-seeded fruit). PTR indicates partial testa removal at the radicle portion of the seeds. Vertical bars represent SE (n = 4). The different letters indicate significant differences at *p* < 0.05 (Duncan’s new multiple range test).

Samaras of *A. tegmentosum* did not germinate after incubation at 15/6 °C and 25/15 °C for 14 weeks. In seeds without the pericarp, germination of 9.7 ± 3.57% and 20.9 ± 8.33% was observed after incubation at 15/6 °C and 25/15 °C for 14 weeks, respectively. At 14 weeks, there was no significant difference between the germination percentage of intact seeds of the two temperature regimes. Seeds with partial removal of the testa germinated to 39.2 ± 7.10 and 70.6 ± 6.65% after 14 weeks at 15/6 °C and 25/15 °C, respectively. At 14 weeks, the germination percentage of seeds with partial removal of the testa was significantly higher at 25/15 °C than at 15/6 °C (Figure 7).

### 2.7. Effect of Washing on Germination

**Figure 8:**
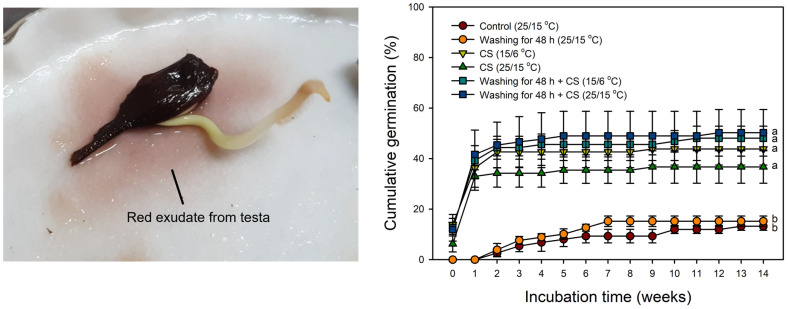
Effect of washing for 48 h on cumulative germination of *Acer tegmentosum* seeds at 15/6 °C and 25/15 °C for 14 weeks. The seeds (pericarp removed) were washed to remove the reddish exudate from the testa. CS indicates cold stratification at 4 °C for 12 weeks. Vertical bars represent SE (n = 4). The different letters indicate significant differences at *p* < 0.05 (Duncan’s new multiple range test).

There was no significant difference between the washed and unwashed seeds with or without cold stratification (Figure 8).

## 3. Discussion

Samaras of *A. tegmentosum* under four temperature regimes showed that no germination occurred within 18 weeks (Figure 4), and, thus, seeds of *A. tegmentosum* could be considered deeply dormant during dispersal. Among the classes of seed dormancy, there is PY facilitated by the water-impermeable covering structures such as seed coat and pericarp [19]. If a dried seed or fruit weight increases by ≥20% due to imbibition, then seeds are considered to have no PY [38]. In our study, the weight of *A. tegmentosum* samaras increased >70% in the imbibition test (Figure 2). Meanwhile, the move-along experiments demonstrated that cold stratification was more efficient in breaking the dormancy of samaras than warm stratification followed by cold stratification (Figure 4). In addition, extending the cold stratification period significantly enhanced the germination of samaras when incubated at 15/6 °C (Figure 5). These results indicate that *A. tegmentosum* seeds have PD at dispersal time and dormancy release is possible only through winter duration under natural conditions. The depth of dormancy and gene expression patterns in seeds are closely related to seasonal temperature changes [39]. The GATA zinc finger transcription factor BME3 is induced during cold stratification and promotes the expression of GA biosynthesis genes *GIBBERELLIN 3 OXIDASE* (*GA3ox1*) and *GIBBERELLIN 20 OXIDASE* (*GA20ox3*) in seeds [21,40]. Several studies also reported that prolonged cold stratification is effective in breaking the dormancy of *Acer* samaras [27,33,41,42,43]. This trait of *Acer* is a strategy adopted to prevent germination in autumn after dispersal from the mother plants [23].

Through the removal of the thin and tough testa, the germination percentage of seeds reached 70% at 14 weeks, which is similar to the final germination percentage of the field experiment (Figure 3 and Figure 7). Thus, it was shown that the removal of the testa around the radicle is effective in promoting the germination of *A. tegmentosum*. In previous studies, removing the testa around the radicle and completely removing the testa were effective in promoting the germination of *Acer* [29,44,45]. Wilson et al. [29] suggested that the testa-imposed dormancy of *A. pensylvanicum* is due to the mechanical resistance of the testa. Recently, Kidd [30] found that ungerminated *A. tataricum* seeds after cold stratification could induce germination by removing the testa around the radicle. On the other hand, we observed the release of a red exudate from the testa (Figure 8). The exudate of *A. ginnala* inhibited the development of lettuce (*Lactuca sativa*) lateral roots [46]. The germination of *Pinus pinea* was enhanced through washing the seeds for 24 h, which resulted in the removal of germination inhibitors such as ABA from the testa [47]. However, in our study, since germination was possible with only partial removal of the testa (Figure 7) and washing for 48 h was not effective in promoting germination (Figure 8), it could be said that the testa-imposed dormancy of *A. tegmentosum* is not due to chemical inhibitors such as ABA or phenolic compounds. It is possible that the testa mechanically restricts the elongation of radicles in *A. tegmentosum*, which indicates that the mechanical resistance of the testa is an important factor in PD of *A. tegmentosum* seeds.

Previously, the mechanical resistance of covering structures was considered to be a type of exogenous dormancy (also known as mechanical dormancy), but in the classification system of Baskin and Baskin [19], mechanical resistance is indicated as a component of PD. Gleiser et al. [45] indicated that *A. opalus* seeds display a slight PD as well as testa-imposed dormancy. The germination of the testa-removed *A. opalus* embryo progressed faster under a 3-month cold stratification period [45]. In our study, when the testa was partially removed, germination increased slowly and was delayed compared with the field experiment, including the winter period (Figure 3 and Figure 7), which indicates that the embryos of *A. tegmentosum* exhibited PD during dispersal. In contrast, GA is known to overcome the mechanical resistance of tissues such as aleurone and testa [48,49]. To overcome the mechanical constraints of seeds with PD, it is necessary to increase the growth potential of the embryo by GA and/or reduce the mechanical resistance of the covering structure [49]. Thus, it could be said that cold stratification not only breaks the PD of *A. tegmentosum* but also helps overcome the mechanical resistance of the testa. However, although it was determined that enzyme expression by GA acts on endosperm weakening in albuminous seeds, the influence of cold stratification on mechanical properties of testa, such as tensile strength, remains unknown [30,49].

Except when the testa was partially removed (Figure 7), the germination percentage of laboratory experiments was lower than that of field experiment (Figure 3, Figure 4, Figure 5, Figure 6, Figure 7 and Figure 8). This phenomenon was also observed in several *Acer* species, including *A. caesium* [41,50]. Gleiser et al. [45] reported that samaras of *A. opalus* showed a relatively low germination percentage after cold stratification for 3 months and suggested that two periods of low-temperature treatments are required under natural conditions. In our study, we observed several differences in environmental factors between laboratory and field experiments. Firstly, temperatures of outdoor stratification may be involved in enhancing the germination of *A. tegmentosum*. In general, temperatures close to 4 or 5 °C are suitable for cold stratification in several species [51,52], but our study showed that lower temperatures (i.e., 1 °C) could affect the dormancy release of *A. tegmentosum* (Figure 5). In terms of light requirements, several germination tests in the laboratory were conducted under the 12L:12D photoperiod, whereas in the field experiment, the samaras were buried in the soil. However, light was not essential for the germination of *A. tegmentosum* samaras after cold stratification for 12 weeks (data not shown). Previously, Zhang et al. [53] reported that the alternating irradiation of red and far-red lights could promote the germination of *A. mono* and suggested the possible involvement of sunflecks under natural conditions. However, since we used PE film as a substitute for canopy and buried the seeds in the soil in our study, light may be excluded from the environmental factors affecting germination.

Results of laboratory experiments showed that the weakening of the testa may be important for dormancy release in *A. tegmentosum* (Figure 7). Various factors (i.e., microbial action, ingestion by vertebrates, weathering, extreme temperatures, fires, and exposure to solar radiation) were suggested for the removal of coat-imposed dormancy under natural conditions [54]. Several researchers provided evidence that the mechanical resistance of covering structures can be weakened by the activity of microorganisms [55,56,57,58]. Delgado-Sánchez et al. [56] reported that fungi such as *Penicillium chrysogenum*, *Phoma* sp., and *Trichoderma koningii* can break the mechanical resistance of the testa in *Opuntia streptacantha* seeds with PD. In addition, soil microbial activity driven by temperature is related to environmental signals and can affect seeds in the soil [59]. On the other hand, although cold stratification at 0 to 10 °C is considered effective in breaking the PD of seeds, below zero temperatures were recorded during winter in the field experiment (Figure 3). Tóth and Garrett [50] documented that stratification at −5 to −10 °C prevented germination in *Acer*, but several studies showed that the freeze–thaw sequence is able to enhance germination in some species. Shibata et al. [60] reported that rapid freezing at −20 °C for 24 h followed by thawing is most effective in reducing the impermeability of seeds and the production of normal seedlings in *Astragalus mongholicus* and argued that ice crystals formed during freezing and melting may help destroy the hard seed coat. However, in order to find answers to the questions on the effect of outdoor stratification, further research should focus on the link between phenology and biomechanics.

In order to break the PD of *A. tegmentosum*, samaras were treated with varying concentrations of exogenous GA_3_ before incubation. However, the application of GA_3_ could not be a substitute for prolonged cold stratification (Figure 5 and Figure 6). Although this result does not correspond with the research of Chen et al. [27], several studies reported that exogenous GA_3_ has no effect on promoting the germination and breaking the dormancy of *Acer* samaras [30,61,62]. However, a combination treatment of stratification and exogenous GA_3_ is effective for promoting the germination of some woody species, e.g., *Sorbus torminalis* [63] and *Symphoricarpos oreophilus* [64]. In contrast, the combination treatment was not as effective as cold stratification in our study (Figure 5 and Figure 6). Previously, the combination treatment was not effective for *A. hyrcanum* samaras and the class of dormancy was classified as deep PD [42]. If GA_3_ fails to promote the germination of intact dispersal units (e.g., achenes, acorns, samaras, etc.) and requires 3–4 months of cold stratification to germinate, it is classified as deep PD [19,20]. In the case of *A. tegmentosum* samaras, cold stratification was required for ≥3 months to break PD (Figure 5) and the application of exogenous GA_3_ and combination treatment had no effect. Overall, it is assumed that dormancy of *A. tegmentosum* seeds are deep PD.

Seasonal timing is important for the survival and reproduction of plants, and among varying environmental factors, temperature serves as the environmental cue for seed germination [65]. Our laboratory experiments demonstrated that the germination of *A. tegmentosum* preferred early spring temperature (15/6 °C) rather than summer temperature (25/15 °C) after cold stratification at 1 and 4 °C (Figure 5 and Figure 6). Interestingly, germination in the field occurred from February to March, and the timing of germination was similar to the results of the laboratory experiment (Figure 3 and Figure 4). It was reported that several *Acer* species such as *A. macrophyllum* [62], *A. rubrum* [66,67], and *A. pensylvanicum* [68] prefer a germination temperature of 15/5 °C or 15 °C rather than high temperatures after cold stratification. The germination of *A. pensylvanicum* was higher at 15/5 °C to simulate a forest stand of April and May in New Brunswick, Canada, after prolonged cold stratification, but germination at 30/20 °C, which is used as standard temperature regimes for germination tests, was lower [68]. Bourgoin and Simpson [68] indicated that 15/5 °C lowered the chilling requirement or induced secondary dormancy due to 30/20 °C, which resulted in the poor germination of *A. pensylvanicum* at 30/20 °C. It is known that the *DELAY OF GERMINATION1* (*DOG1*) gene is involved not only in primary dormancy such as PD but also in secondary dormancy due to high temperature [69].

However, the removal of the pericarp showed that germination is even possible at 25/15 °C (Figure 7). In addition, although it was almost impossible to germinate the samaras at 25/15 °C after cold stratification (Figure 5 and Figure 6), seeds germinated at 25/15 °C and as much as 15/6 °C after cold stratification (Figure 8). These results indicate that suppression of germination by 25/15 °C is caused by the impact of the pericarp. This phenomenon can also be found in popular crops such as spinach (*Spinacia oleracea*) and sunflower (*Helianthus annuus*). The germination of spinach fruits was inhibited at 35 °C, but this was not the case when the pericarp was removed [70]. Suganuma and Ohno [70] described the possibility that the pericarp can limit oxygen uptake at high temperatures. Gay et al. [71] also suggested that the pericarp and testa of sunflower inhibited germination at high temperatures, indicating the same reason. Thus, it could be said that the pericarp suppresses the germination of *A. tegmentosum* at 25/15 °C. In the previous study, it was claimed that the pericarp of *A. pseudoplatanus* inhibits oxygen uptake to the embryo [44]. That being the case, further investigation is needed to identify the reason behind this trait in *A. tegmentosum*.

Bourgoin and Simpson [68] suggested that the seeds of *A. pensylvanicum* may have adapted to germination at cooler temperatures. Seedling emergence during early spring is known to have benefited their survival. For example, the early-emerging cohorts of *A. mono*—one of the earliest germinating species in the hardwood forest of northern Japan—showed a higher seedling survival rate than the late-emerging cohorts; seedlings that emerge early experience better growing conditions such as light or are benefitted by the relatively lower pathogen and predator activity during the early, cooler spring [72]. *A. platanoides* germinates at very low temperatures after chilling, allowing it to be successfully established before the soil dries in summer [23]; this is particularly beneficial for the perennial species of the Mediterranean and other arid regions with dry summers [73]. Short growing periods due to late emergence can be problematic during drought as the root system does not absorb sufficient quantities of water [74] whereas germination at lower temperatures facilitate better seedling establishment [75]. Kanazashi et al. [76] attributed the germination of *A. pycnanthum* in early spring to avoiding competition with neighbors. Hence, the behavior of *A. tegmentosum*, which germinates at 15/6 °C and whose germination is inhibited at 25/15 °C, may indicate a temporal niche differentiation to avoid competition with neighbors in its vicinity.

Our study demonstrated that PD, including the mechanical resistance of the testa, could be broken by the low temperature of winter; the impact of the pericarp allowed germination to occur during early spring. Each covering structure plays an important role in determining the early life history of *A. tegmentosum*, suggesting that the testa and pericarp in *A. tegmentosum* are adapted to the climate of Northeast Asia. Based on previous reports, we observed that the optimum temperature for germination after cold stratification varies with different *Acer* species. *A. morrisonense*, endemic to Taiwan and belonging to the section *Macrantha*, was able to germinate at 25/15 °C after cold stratification [27]. *A. tegmentosum* and *A. morrisonense* belong to the same clade; both share the same geographic distribution [77] but differ in their germination traits after cold stratification. Four Caryophyllaceae species, which share a close phylogenetic and geographic relationship, differed in the timing and location of seedling emergence; this was attributed to their habitat preference rather than factors such as phylogeny [35]. Thus, differences in the germination traits of *A. tegmentosum* and *A. morrisonense* could be attributed to their adaptation to habitat owing to environmental selection pressures. However, further investigations focusing on the factors affecting the germination temperature of other *Acer* species are required to understand the germination ecophysiology in *Acer*.

## 4. Materials and Methods

### 4.1. Collection and Characteristics of Samaras

Mature samaras (single-seeded fruits) of *A. tegmentosum* were collected from the Hantaek Botanical Garden in Korea (37°05′40.4″ N, 127°24′19.3″ E) during October 2020 (Table 1). Except for phenology experiments, samaras were dried for approximately 2 weeks at room temperature. Dried samaras were stored at 0 °C with silica gel until further use. Length, width, and thickness of wingless samaras were examined using a metric vernier caliper; 100-seed weight of wingless samaras (g) was measured using an electronic scale (PAG213, OHAUS Corp., Parsippany, NJ, USA). The internal structure of samaras and embryo shape were observed using a USB digital microscope (AM3111 Dino-Lite premier, ANMO Electronics Co., Hsinchu, Taiwan). To determine the imbibition of wingless samaras, wingless samara weight was measured for 48 h, as described [78]. The increase in fruit weight was measured using the formula:

%Ws = [(Wh − Wi)/Wi] × 100

where Ws = increase in seed weight, Wh = weight of seeds after a given interval of imbibition, and Wi = initial seed weight.

### 4.2. Phenology of Germination and Seedling Emergence in a Field

An experimental garden located at Andong National University in Korea (36°32′40.5″ N, 128°48′2.81″ E) was used for phenology studies. To block out sunlight, and to mimic the canopy of a deciduous forest, a shade netting made of polyethylene (PE) film was installed at the garden during the experiments. In winter, all the deciduous trees in the habitat lose their leaves, so the installed shade netting was temporarily removed. Samaras were used for the experiment after removal of all of the wings. Four replicates of 20 samaras were buried at 3.5 cm depth in plastic pots filled with sand. All of the plastic pots were buried in the ground. Germination and seedling emergence were recorded at weekly intervals. Samaras were taken out to check for germination and considered to have germinated when radicles emerged ≥2 mm from the pericarp. Seedling emergence was recorded when the shoot emerged above the ground. Soil temperature at a 3 cm depth was measured every 30 min with a data logger (Watch Dog Model 1425, Spectrum Technologies, Inc., Plainfield, IL, USA); daily average, maximum, and minimum soil temperatures were calculated.

### 4.3. Germination Test in the Laboratory

Germination tests in the laboratory were conducted using incubators set to constant temperature regimes of 1 and 4 °C and alternating temperature regimes of 15/6 °C, 20/10 °C, and 25/15 °C. These temperature regimes represent the seasonal conditions in temperate regions [20,38]. The daily photoperiod of the incubator was 12L:12D, and light (PPFD 10.2 ± 0.85 μmol·m^−2^·s^−1^) was provided by a cool white fluorescent lamp. Four replicates of 20 samaras (or seeds) were incubated under each treatment group. All samaras used had their wings removed. The samaras were soaked with 1000 mg∙L^−1^ benomyl for 12 h for sterilization and sown with distilled water in 90 mm dia × 15 mm deep Petri dishes with two layers of filter paper. The Petri dishes were sealed with parafilm. The samaras (or seeds) were considered to have germinated when radicles emerged ≥2 mm from the pericarp (or testa). Germination was recorded at weekly intervals except in the case of move-along experiments.

### 4.4. Move-Along Experiments

Based on Baskin and Baskin [38], each treatment consisted of control (4 °C, 15/6 °C, 20/10 °C, and 25/15 °C) and two sequences that simulated seasonal temperature changes under natural conditions: (a) Move A: 4 °C (winter) for 12 weeks → 15/6 °C (early spring) for 4 weeks → 20/10 °C (late spring) for 4 weeks → 25/15 °C (summer) for 4 weeks; (b) Move B: 20/10 °C (early autumn) for 4 weeks → 15/6 °C (late autumn) for 4 weeks → 4 °C (winter) for 12 weeks → 15/6 °C (early spring) for 4 weeks. Move B was designed to determine whether the autumn period influences breaking seed dormancy when samaras of *A. tegmentosum* are dispersed from the mother plants. Germination was recorded every 2 weeks.

### 4.5. Effect of Cold Stratification on Germination

Samaras were subjected to 1 and 4 °C for 0, 4, 8, and 12 weeks. After completion of each cold stratification treatment, the respective samaras’ samples were incubated at 15/6 °C and 25/15 °C for 14 weeks.

### 4.6. Effect of Exogenous GA_3_ on Germination

Samaras were treated with solutions of 0, 10, 100, and 1000 mg∙L^−1^ GA_3_ for 24 h. Distilled water was used as the control. The samaras were incubated at 15/6 and 25/15 °C for 14 weeks. A combination treatment involving 1000 mg∙L^−1^ GA_3_ for 24 h followed by cold stratification at 4 °C for 12 weeks was also performed for samaras. After the completion of combination treatment, the samaras were incubated at 15/6 °C and 25/15 °C.

### 4.7. Effect of Pericarp and Testa on Germination

This experiment was performed to investigate the effect of the pericarp and mechanical resistance of the testa on germination of *A. tegmentosum*. The treatment group consisted of samaras (control), seeds (pericarp removed), and seeds with the testa around the radicle removed (PTR, partial testa removal). Each treatment group was incubated at 15/6 °C and 25/15 °C for 14 weeks.

### 4.8. Effect of Washing on Germination

Washing of seeds under running tap water simulates the effect of natural rainfall on the dormancy release of *A. tegmentosum*. Seeds with the pericarp removed from the samara were used. The experiment consisted of six treatments: (a) control (incubated at 25/15 °C); (b) washing for 48 h (incubated at 25/15 °C); (c) cold stratification (incubated at 15/6 °C); (d) cold stratification (incubated at 25/15 °C); (e) washing for 48 h followed by cold stratification (incubated at 15/6 °C); (f) washing for 48 h followed by cold stratification (incubated at 25/15 °C). In each treatment group, the seeds were incubated for 14 weeks. For cold stratification, the seeds were subjected to 4 °C for 12 weeks.

### 4.9. Statistical Analyses

Statistical analyses were performed using SAS 9.4 (SAS Institute, Cary, NC, USA) and SigmaPlot 10.0 (SPSS Inc., Chicago, IL, USA). To analyze the results of each treatment group, either ANOVA followed by Duncan’s new multiple range test (*p* < 0.05) or *t*-test was used.

## 5. Conclusions

The seeds of *A. tegmentosum* have deep physiological dormancy when dispersed from the mother plant, and the dormancy is broken through prolonged cold stratification at 1 and 4 °C. In addition, the samaras of *A. tegmentosum* have water-permeable covering structures composed of testa and pericarp. It was possible to break the dormancy just by removing the tough testa of the radicle without cold stratification or exogenous gibberellic acid (GA_3_) treatments. Therefore, the testa exhibits mechanical resistance preventing radicle elongation. Our results from laboratory and field experiments show that the samaras germinate at early spring temperatures (approximately 15/6 °C) after the prolonged winter period. This germination behavior of *A. tegmentosum* is attributed to the effect of the pericarp and indicates several benefits to the seedling survival of *A. tegmentosum*. The covering structures composed of testa and pericarp of *A. tegmentosum* play an important role in determining the early life history under the climate of Northeast Asia. Our results can be utilized for a seed propagation protocol of *A. tegmentosum* and can also be used to indirectly contribute to reducing overexploitation through commercial cultivation.

## Figures and Tables

**Table 1:** Characteristics of *Acer tegmentosum* samaras (single-seeded fruit) used in this study.

Collection Location	Collection Date	Length (mm)	Width (mm)	Thickness (mm)	100 Seed Weight (g)
Hantaek Botanical Garden (37°05′40.4″ N, 127°24′19.3″ E)	3–19 October 2020	10.4 ± 0.12 ^z^	5.2 ± 0.08	2.1 ± 0.04	3.9 ± 0.03

^z^ Values are expressed as mean ± SE.

## Data Availability

The original contributions presented in this study are included in the article. Further inquiries can be directed to the corresponding authors.
